# Partially overlapping sensorimotor networks underlie speech praxis and verbal short-term memory: evidence from apraxia of speech following acute stroke

**DOI:** 10.3389/fnhum.2014.00649

**Published:** 2014-08-25

**Authors:** Gregory Hickok, Corianne Rogalsky, Rong Chen, Edward H. Herskovits, Sarah Townsley, Argye E. Hillis

**Affiliations:** ^1^Department of Cognitive Sciences, Center for Language Science, Center for Cognitive Neuroscience and Engineering, University of CaliforniaIrvine, CA, USA; ^2^Department of Speech and Hearing Science, Arizona State UniversityTempe, AZ, USA; ^3^Department of Diagnostic Radiology and Nuclear Medicine, University of MarylandBaltimore, MD, USA; ^4^Department of Neurology, Johns Hopkins University School of MedicineBaltimore, MD, USA; ^5^Department of Physical Medicine and Rehabilitation, Johns Hopkins University School of MedicineBaltimore, MD, USA; ^6^Department of Cognitive Science, Johns Hopkins UniversityBaltimore, MD, USA

**Keywords:** apraxia of speech, speech production, motor control, aphasia, language, stroke, short term memory

## Abstract

We tested the hypothesis that motor planning and programming of speech articulation and verbal short-term memory (vSTM) depend on partially overlapping networks of neural regions. We evaluated this proposal by testing 76 individuals with acute ischemic stroke for impairment in motor planning of speech articulation (apraxia of speech, AOS) and vSTM in the first day of stroke, before the opportunity for recovery or reorganization of structure-function relationships. We also evaluated areas of both infarct and low blood flow that might have contributed to AOS or impaired vSTM in each person. We found that AOS was associated with tissue dysfunction in motor-related areas (posterior primary motor cortex, pars opercularis; premotor cortex, insula) and sensory-related areas (primary somatosensory cortex, secondary somatosensory cortex, parietal operculum/auditory cortex); while impaired vSTM was associated with primarily motor-related areas (pars opercularis and pars triangularis, premotor cortex, and primary motor cortex). These results are consistent with the hypothesis, also supported by functional imaging data, that both speech praxis and vSTM rely on partially overlapping networks of brain regions.

As effortless as it seems, articulation of speech requires orchestration of an incredibly complex motor system: rapid and fine-tuned timing of movements of the lips, tongue, jaw, soft palate, vocal folds, laryngeal muscles, and respiratory muscles. These movements must all occur in the correct order for the word to be pronounced correctly. A breakdown in this motor plan or sequence of articulatory movements results in variable substitutions, deletions, transpositions, and insertions of speech sounds, known as apraxia of speech (AOS). AOS is a speech disorder characterized by slowed speech rate, sound distortions, phoneme substitutions, and trial and error speech attempts; speech difficulties increase with increased length and complexity of utterances (Graff-Radford et al., 2014). The nature of the disorder, as a motor disorder or a linguistic disorder or disorder of phonological short-term memory, has been controversial. But this controversy may be poorly framed. Motor planning of speech articulation, some linguistic processes, and some aspects of phonological short-term memory (e.g., articulatory rehearsal) may all depend on at least some of the same neural mechanisms. That is, all of these functions may depend on a complex network of neurons synapsing in the right order, supporting the accurate maintenance of the sequence of (planned or actual) movements of the muscles of the speech articulation, which are instantiated as a sequence of phonemes, syllables, words, or digits. It is not clear that it makes sense to ask if breakdown in one behavioral manifestation of this process is “caused by” breakdown in another behavioral manifestation of the same underlying neural process. By hypothesis, the behavioral impairments reflect the disruption of the underlying neural mechanism. Depending on the complexity of the behavioral task, any of these tasks (e.g., articulation of an utterance, a linguistic task, rehearsal, recall of digits) will engage other cognitive/neural processes as well, and thus may also be sensitive to disruption in other neural mechanisms.

The underlying anatomy of speech praxis (i.e., location of lesions that result in AOS) has also been controversial, with different stroke-based studies reporting maximal lesion overlap in (or significant associations with) different regions including the anterior insula (Dronkers, 1996), Broca’s area (Hillis et al., 2004; Richardson et al., 2012), and premotor cortex/precentral gyrus (Graff-Radford et al., 2014). Studies of AOS in neurodegenerative disease also implicate premotor cortex and, in addition, the supplementary motor area (SMA; Josephs et al., 2006). Taken together, these studies suggest that there is not a single area that is responsible for planning and orchestration speech articulation, but a *network* of regions functioning together. Such a network view is consistent with models of speech motor control, which implicate sensorimotor networks including a constellation of motor (Broca’s area, premotor cortex, primary motor cortex, SMA), sensory (auditory and somatosensory cortex), and sensorimotor integration areas (posterior parietal lobe/area Spt and cerebellum) (Terband et al., 2009; Golfinopoulos et al., 2010; Hickok et al., 2011; Houde and Nagarajan, 2011; Hickok, 2012).

Meanwhile, work on the neural basis of verbal short-term memory (vSTM), predominantly based on functional imaging methods, has identified some of the same regions including portions of Broca’s area, the anterior insula, and premotor cortex (Awh et al., 1996; Smith and Jonides, 1997; Chein et al., 2002; Hickok et al., 2003; Buchsbaum and D’Esposito, 2008; Chein and Fiez, 2010). As mentioned, this apparent overlap is not unexpected as aspects of motor speech (specifically, articulatory rehearsal) constitute an important component of vSTM (Baddeley, 1992). Indeed, some theorists have explicitly proposed that vSTM is an emergent property—a kind of evolutionary exaptation—of the speech production system (Hickok et al., 2003; Postle, 2006; Buchsbaum and D’Esposito, 2008; Buchsbaum et al., 2011). Thus, there are reasons to predict some degree of overlap in the neural systems underlying vSTM and AOS. Nevertheless, the relation between vSTM and AOS has not been fully elucidated.

The aim of the present study is two-fold. The first is to investigate the neural regions critical for planning and programming of speech articulation by evaluating AOS and associated areas of tissue dysfunction using Magnetic Resonance Imaging (MRI) with perfusion data in a large sample of stroke patients in the *acute* stage. This approach provides a measure of total tissue dysfunction (regions of infarct and low blood flow beyond the infarcted zone) and is not contaminated by compensatory processes—neural or behavioral—that can complicate interpretation of chronic stroke data. The second is to investigate the neural regions critical for vSTM in the same acute stroke dataset and its relation to AOS. There is reason to believe that speech praxis and vSTM have a partially, but not completely, overlapping neural basis (Baddeley, 1992; Hickok et al., 2003; Buchsbaum and D’Esposito, 2008). While AOS is thought to predominantly implicate motor articulation systems—those that one would expect to be involved in rehearsal—vSTM is comprised of a broader network including some form of a phonological store (Baddeley, 1992; Buchsbaum and D’Esposito, 2008; Buchsbaum et al., 2011). Previous work suggests that AOS is associated with a network of regions, rather than a single region, which partially overlaps with the network associated with vSTM deficits in the same patients (Awh et al., 1996; Smith and Jonides, 1997; Chein et al., 2002; Hickok et al., 2003, 2011; Buchsbaum and D’Esposito, 2008; Terband et al., 2009; Golfinopoulos et al., 2010; Chein and Fiez, 2010; Houde and Nagarajan, 2011; Hickok, 2012).

## Methods

### Participants

We enrolled a series of 76 acute stroke patients within 24 h of admission to the hospital for acute ischemic predominantly left hemispheric stroke. Exclusion criteria included the following: patients with purely brainstem or cerebellar stroke; non-native speaker of English; known uncorrected hearing or visual loss; reduced level of consciousness, intubation, or ongoing intravenous sedation; prior neurological disease (including previous stroke); hemorrhage on initial imaging, contraindication to MRI (e.g., implantation of any ferromagnetic metal; pregnancy; severe claustrophobia; allergy to Gadolinium contrast; or moderate to severe renal failure (glomerular filtration rate < 60; contraindication for Gadolinium)). We obtained informed consent from all participants with adequate comprehension, or from their identified decision-maker for those with impaired comprehension. This study was approved by the Johns Hopkins Institutional Review Board.

The participants were age 29–85 years old (mean 58.0; s.d. 12.9), with 4–20 years of education (mean 13.9; s.d. 3.4); 35 (46.1%) were female. The Western Aphasia Battery-Revised (WAB-R; Kertesz, 2012), Aphasia Quotient (AQ) ranged from 12–100, with a mean of 86.9 (s.d. 19.5). National Institutes of Health Stroke Scale (NIHSS) score was recorded in 38 patients (including 10 with AOS), and ranged from 0–24, with a mean of 6.65 (SD 5.5) at the time of admission. Five of the patients (all without AOS) had received intravenous tissue plasminogen activator (tPA) prior to the MRI or speech and language testing. None of the participants were completely mute, but one produced no intelligible words. A total of 54 patients had aphasia as classified by the WAB-R (AQ < 93.8).

### Procedure

Participants were administered a battery of language tasks that took approximately 1.0–2.0 h to administer, depending on stroke severity. This battery included standardized tasks, including the Apraxia Battery for Adults (ABA; Dabul, 2000) and the WAB-R, as well as non-standardized digit span tasks (described below) and additional naming, reading, spelling, and comprehension tests used our Stroke Cognitive Outcomes and REcovery (SCORE) lab and the Multicenter Aphasia Recovery Center (MARC) (not relevant to this study).

A trained technician administered the tests. The ABA involves structured speech tasks (e.g., repetition of words of increasing length), as well as obtaining a variety of speech samples (oral reading, serial speech, discourse), which are scored for characteristics of AOS (e.g., “exhibits abnormal prosodic features”, “exhibits visible/audible searching”, “exhibits numerous and varied off-target attempts at the word”, “exhibits awareness of errors and inability to correct them”, and “exhibits expressive-receptive gap”). The speech samples were recorded and reviewed by two speech-language pathologists who came to agreement on the final scores. We defined AOS as ≥3 of four abnormal scores on the ABA subtests of Words of Increasing Length A and B (which scores an increase in articulatory errors with increased word length for short and longer words/phrases, respectively), Repeated Trials (which scores variability in errors in repetition of the same polysyllabic words), and Inventory of Articulation (which scores characteristics of AOS). Absence of AOS was defined as 0 or 1 abnormal scores on these four subtests. Cases of two of four abnormal scores were considered “indeterminate” and were not included in the study. We used a dichotomous classification of AOS, because we are not aware of a well-validated and reliable objective measure of severity of AOS. A patient who has more characteristics of AOS does not necessarily have more severe AOS, for example. (In fact, the most severely apractic patients are mute or almost so, and have fewer characteristics). Even more errors on polysyllabic words does not indicate that the patient has more severe AOS (again because the most severe patients are almost mute and produce few “errors”).

#### Verbal short-term memory (vSTM) task

vSTM was assessed with a forward digit span task. Participants heard series of numbers, of increasing length, which they repeated. They were asked to both point to the correct numbers, and say them aloud (if they could speak). The technician recorded whichever response was more accurate if there was a discrepancy between spoken and pointing response. Occasionally, a patient with AOS would point to digits he or she could not say, and these were recorded as the response.

Participants received two trials at each list length, beginning with 2-items per series. Testing continued until the participant failed both items at a given span length, and span was calculated as the maximum list length for which the participant was successful on at least one trial. Impaired digit span was defined as a forward digit span <5.

#### Magnetic Resonance Imaging (MRI)

Patients underwent MRI within 48 h of being admitted to the hospital (within 24 h of behavioral testing). The MRI protocol included the following: Axial T2 and Fluid Attenuated Inversion Recovery (FLAIR; to evaluate for old lesions), Diffusion-Weighted Imaging (DWI) to identify the site of acute infarct; susceptibility weighted imaging (SWI; to evaluate for hemorrhage); and high-resolution magnetization-prepared rapid acquisition with gradient echo (MPRAGE) to register to the MNI atlas; and dynamic-susceptibility contrast echo-planar Perfusion Weighted Imaging (PWI). For PWI, 20 cc of Gadolinium was power-injected at 5 cc/s. All scans were acquired parallel to the anterior commissure-posterior commissure line.

**Figure 1:**
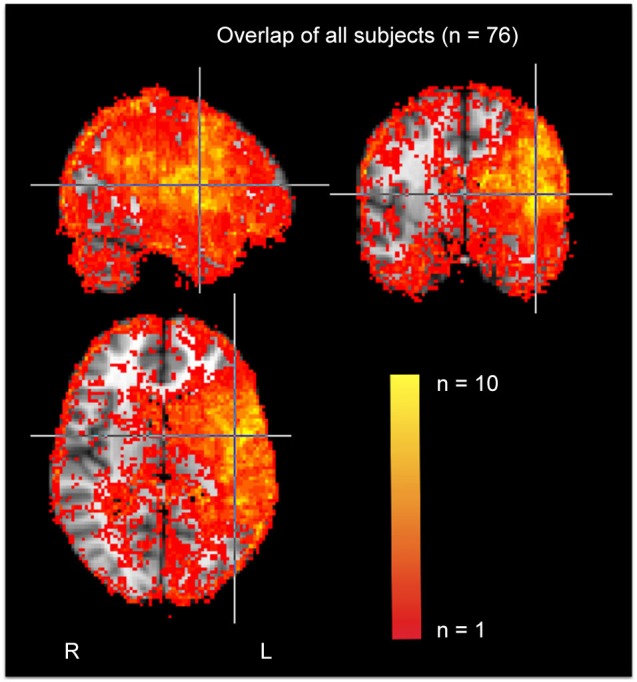
**Group map of infarcted/hypoperfused voxels in the entire sample**.

To identify areas of dense ischemia/infarct, the areas that were bright on DWI and dark on apparent diffusion coefficient (ADC) were manually drawn using MRICron. For PWI scans, dysfunction was indicated by >4 s delay in time to peak (TTP) arrival of Gadolinium in a voxel, compared to the homologous voxel in the right hemisphere. Four seconds delay in TTP corresponds to dysfunctional tissue as defined by positron emission tomography (PET; Sobesky et al., 2004; Zaro-Weber et al., 2010). The segmented “lesion” was dysfunctional tissue that was either infarcted on DWI/ADC and/or hypoperfused on PWI. In some acute stroke cases the infarct is larger than the area of hypoperfusion (when there has been immediate reperfusion), and in others, the area of hypoperfusion extends beyond the infarct. A group map of affected voxels in our sample is provided in Figure 1.

#### Lesion-deficit analysis procedures

Voxel-based lesion symptom mapping (VLSM; Bates et al., 2003) implemented in Matlab (Mathworks, Inc.) was used amongst the 76 patients to identify voxels for which a *t*-test indicates that patients with dysfunction in that voxel perform significantly different than patients who do not have dysfunction in that voxel. “Dysfunction” was determined using both DWI and the PWI data, thus including both densely infarcted and hypoperfused tissue. VLSMs were conducted for AOS and forward digit span measures, respectively. A voxel-wise significance threshold of *p* < 0.01 was used, and then multiple comparisons were controlled for using a cluster size-based permutation method (1000 permutations), such that significant voxel clusters that were larger than 95% of the significant clusters in the random permutations passed threshold. Variance due to functional lesion size (i.e., number of voxels with infarct and/or significant hypoperfusion) was regressed out of both VLSM analyses via an Analysis of Covariance (ANCOVA). The resulting statistical maps are visualized overlaid upon the MNI152 standard-space T1-weighted template brain using FMRIB Software Library’s (FSL) View software (Smith et al., 2004).

## Results

### Behavioral results

There were 17 patients with AOS and 59 patients without AOS using the above criteria for AOS. There were no significant differences by independent *t*-tests between those with and without AOS in age (57.8 ± SD 14.2 vs. 58.0 ± 12.6; *t* = 0.05; *p* = 0.96) or stroke severity measured by NIHSS (mean 6.5 ± 5.8 vs. 6.7 ± 5.5; *t* = 0.1; df35; *p* = 0.92). There were 15 patients with impaired digit span, and 61 without impaired digit span. There was no difference between those with and without impaired digit span in age (mean 59.8 vs. 57.5 years; *t* = 0.62; df74; *p* = 0.54) or stroke severity (NIHSS = 6.6 ± 5.5 vs. 6.7 ± 5.5; *t* = 0.1; df35; *p* = 0.92).

There were 12 individuals with both AOS and impaired vSTM; five with AOS with normal vSTM; and three with reduced vSTM but no AOS. There was a strong association between AOS and vSTM (*χ*^2^ = 35.7 ; *p* < < 0.0001). All 17 patients with AOS also had aphasia (defined as AQ on the WAB of <93.8); however, 37 patients had aphasia without AOS. Likewise, all 15 patients with impaired vSTM and aphasia; however, there were 39 patients with aphasia with normal vSTM. Aphasia was significantly (but weakly) associated with AOS (*χ*^2^ = 7.0 ; *p* = 0.01) and vSTM (*χ*^2^ = 5.3 ; *p* = 0.02).

To further assess the relation between AOS and vSTM behaviorally, and to make sure the association could not simply be attributed to an association with a third variable, large infarcts, we carried out an ANCOVA using AOS as a two-level factor (AOS+ vs. AOS−), lesion volume as a covariate to factor out the effects of lesion size, and digit span score as the dependent variable. The adjusted means reflected worse digit span performance in those patients *with* AOS (AOS+, adjusted mean = 3.41) compared to patients *without* AOS (AOS−, adjusted mean = 6.54). This difference proved to be highly reliable in the ANCOVA (*F*_(1,73)_ = 33.92, *p* < 0.001). Thus, AOS is associated with reduced digit span.

### Imaging results

No significant voxels were found in the right hemisphere. All results reported are for left hemisphere structures.

**Figure 2:**
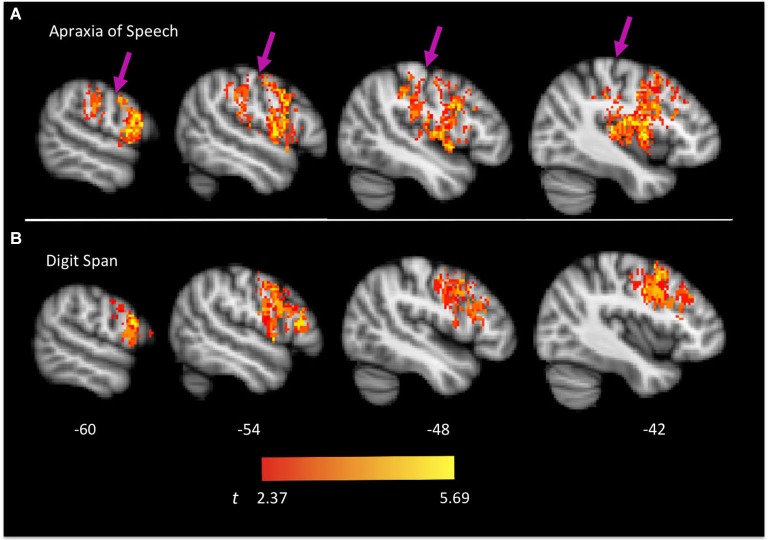
**Group map of voxels significantly related to apraxia of speech (A) and impaired digit span performance (B)**. Left hemisphere is shown. Arrows indicate the central sulcus.

**Figure 3:**
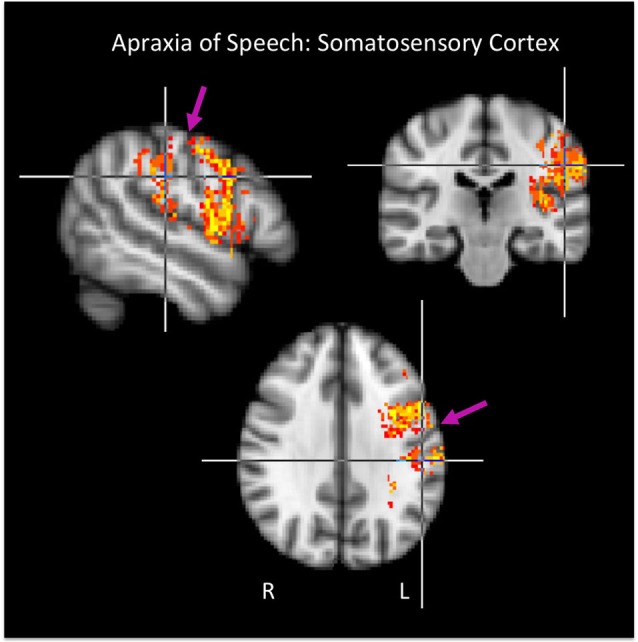
**Group maps showing voxels in somatosensory cortex significantly related to apraxia of speech**. Arrows indicate the central sulcus.

Brain regions associated with AOS are presented in Figures 2A, 3 and include the pars opercularis (posterior sector of Broca’s area), premotor cortex, the anterior and posterior insula, pre-central gyrus, post-central gyrus, post-central sulcus, posterior parietal/parietal operculum, and auditory cortex/parietal operculum (although the analyses clearly turn up voxels in primary auditory cortex, the close proximity to the parietal operculum suggests extreme caution in localizing a real effect to auditory cortex). Table 1 provides a list of involved areas based on the Julich probabilistic cytoarchitectonic atlas and Talairach atlas provided in FSL (Talairach and Tournoux, 1988; Lancaster et al., 2000, 2007; Eickhoff et al., 2005, 2006, 2007). Dysfunction in voxels in these regions results in a significantly higher incidence of AOS compared to when these voxels are non-ischemic (non-infarcted and normally perfused).

**Table 1:** **Anatomical locations containing significant voxels in the apraxia of speech and digit span VLSMs, respectively**.

	Apraxia of speech	Digit span
Frontal	L inferior frontal gyrus (44)	L inferior frontal gyrus (44/45)
	L middle frontal gyrus (9)	L middle frontal gyrus (9)
	L pre-central gyrus (4/6)	L pre-central gyrus (4/6)
Parietal	L inferior parietal lobule (40)	—
	L post-central gyrus (3/1/2/43)	—
	L supramarginal gyrus (40)	—
Temporal	L transverse temporal gyrus (41)	—
Other	L insula (13)	—

*Approximate Brodmann areas of implicated voxels are in parentheses*.

Brain regions associated with impaired digit span are presented in Figure 2B and include predominantly frontal regions: Broca’s area (both the pars opercularis and portions of the pars triangularis), premotor cortex, and precentral gyrus. Table 1 provides a list of involved areas based on the Julich probabilistic cytoarchitectonic atlas and Talairach atlas provided in FSL (Talairach and Tournoux, 1988; Lancaster et al., 2000, 2007; Eickhoff et al., 2005, 2006, 2007). Dysfunction in voxels in these regions results in significantly lower digit span scores compared to when these voxels are normally perfused.

**Figure 4:**
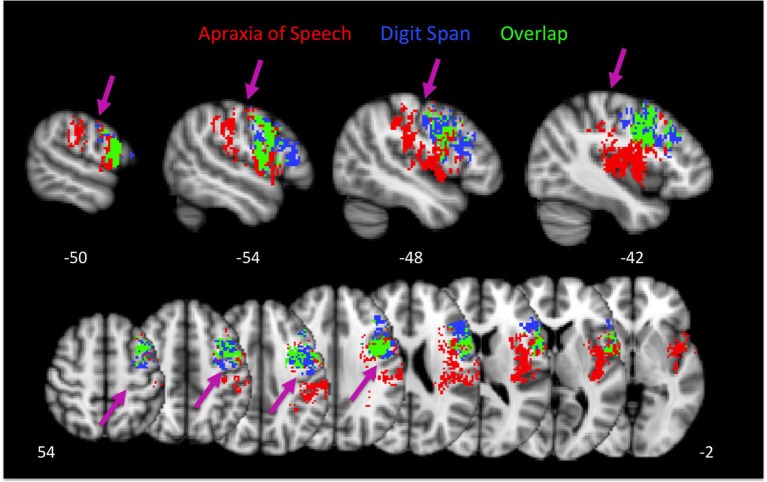
**Group maps showing the relation between voxels significantly related to apraxia of speech and impaired digit span performance**. Arrows indicate the central sulcus.

As is evident, there is substantial overlap in the brain regions associated with AOS and impaired digit span. Regions of overlap include the pars opercularis portion of Broca’s area, premotor cortex, and motor cortex in the precentral gyrus. Regions implicated in AOS but not impaired digit span include the insula, somatosensory cortex in the post central gyrus, the temporal-parietal junction, and auditory cortex. Regions implicated in impaired digit span but not AOS include the pars triangularis portion of Broca’s area. Figure 4 displays the relation between regions implicated in AOS and impaired digit span.

## Discussion

Several findings emerged from this study. Our results are consistent with the hypothesis that both speech praxis and vSTM rely on *networks* of regions, not single areas (see also, Trupe et al., 2013). For speech praxis a *sensorimotor* network was identified that included not only a constellation of motor-related areas (primary motor cortex, pars opercularis; premotor cortex, insula) but also sensory-related areas (primary somatosensory cortex, secondary somatosensory cortex, parietal operculum/auditory cortex). For vSTM a more focal network emerged that included primarily motor-related areas, pars opercularis and pars triangularis, premotor cortex, and primary motor cortex. The networks underlying speech praxis and vSTM overlap in the pars opercularis, premotor, and motor cortex, an overlap that is reflected in behavior: patients with AOS have reduced digit spans compared to patients without AOS.

### Relation between disrupted networks associated with AOS and speech motor control models

The network organization of speech production revealed by infarct/hypoperfusion patterns in AOS following acute stroke is consistent with current behavioral data and neurocomputational models of speech motor control (Terband et al., 2009; Golfinopoulos et al., 2010; Hickok et al., 2011; Houde and Nagarajan, 2011; Hickok, 2012). It is well-established that motor control generally and speech motor control specifically is dependent on sensorimotor integration (Wolpert et al., 1995; Guenther et al., 1998; Houde and Jordan, 1998). With respect to speech, there is strong evidence for both auditory-motor (Houde and Jordan, 1998; Larson et al., 2001; Houde et al., 2002; Heinks-Maldonado et al., 2006; Tourville et al., 2008) and somatosensory-motor interaction (Tremblay et al., 2003; Nasir and Ostry, 2006). This work demonstrates that the targets for speech acts are sensory in nature (Guenther et al., 1998; Perkell, 2012) and most computational models assume that sensorimotor circuits implement one or another form of sensory-guided feedback control of motor speech actions (Tourville et al., 2008; Golfinopoulos et al., 2010; Hickok et al., 2011; Houde and Nagarajan, 2011; Hickok, 2012). In this context, it is not surprising to find that a motor speech deficit, such as AOS, implicates sensory systems.

It is interesting that AOS strongly implicates somatosensory cortex, both SI and SII, in our study. Previous smaller-scale studies of AOS have indicated this possibility (McNeil et al., 1990), but somatosensory regions have not emerged from larger-scale investigations (Dronkers, 1996; Hillis et al., 2004; Trupe et al., 2013). Somatosensory cortex *has* been implicated in speech production error patterns in aphasia, however. Schwartz et al. (2012) report a recent large-scale study of chronic aphasia (*N* = 106) in which phonological errors in naming were found to be significantly related to damage in somatosensory cortex including both SI and SII, with the largest cluster including the post-central gyrus. The sample of 106 patients included 23 patients with AOS. When these patients were removed from the analysis, the distribution of implicated regions shifted posteriorly and centered on the supramarginal gyrus rather than the post-central gyrus (still likely involving SII, however) and also included *auditory-*motor area Spt, which occupies the posterior Sylvian region at the parietal-temporal boundary (including posterior parietal operculum and planum temporale) (Hickok et al., 2003, 2009; Buchsbaum et al., 2011). The pattern of *sensory* cortex damage associated with AOS in the present study more closely resembles the pattern Schwartz et al. report for their whole sample (including the AOS patients): we did not observe substantial involvement of the posterior Sylvian region. These patterns of results hint at a hierarchical organization of sensorimotor circuits (Hickok, 2012) in which the somatosensory system participates in a lower level of motor control, consistent with the more motoric nature of AOS, and in which the auditory-motor system participates in a higher level of motor control, consistent with non-apraxic (e.g., “phonological”) speech errors typical of conduction aphasia and other posterior, fluent aphasias (Hillis, 2007). Indeed, the lesions associated with conduction aphasia overlap with auditory-motor area Spt (Buchsbaum et al., 2011).

### Relation between vSTM network and functional imaging-based models of vSTM

We found a more restricted set of regions involved in vSTM deficits compared to those implicated in AOS. Whereas AOS implicated both motor and sensory regions, vSTM deficits were associated primarily with motor speech areas. Given behavioral (Baddeley, 1992) and functional-anatomic models (Awh et al., 1996; Smith and Jonides, 1997; Buchsbaum et al., 2005; Baldo and Dronkers, 2006; Postle, 2006) of vSTM, the involvement of motor areas is predicted as vSTM involves an articulatory rehearsal component. But vSTM also involves a sensory or “storage” component, leading one to expect, also, the involvement of posterior, sensory-related regions (Paulesu et al., 1993; Jonides et al., 1998; Wilson, 2001; Ruchkin et al., 2003; Buchsbaum and D’Esposito, 2008). Closer consideration, however, suggests a possible explanation for why sensory-regions are not implicated in the present study. In models of vSTM the sensory or storage component is a passive store that maintains information over a limited temporal window of approximately 2 s (Baddeley, 1992). Thus, without the articulatory rehearsal component, span would drop to very low list lengths (~2 items in a span task that involves presenting items 1 every second). It follows that whether the patients were classified as impaired vSTM or not (whether their digit span was <5 or ≥5) depended primarily on the status of articulatory rehearsal. Thus, our findings for the neural correlates of vSTM deficits can be interpreted as primarily reflecting impairments of articulatory rehearsal, which implicates motor-related regions. We also may not have had sufficient number of patients with tissue dysfunction in posterior storage-related areas to have the power to detect the association with impaired vSTM.

## Conclusions

The present study suggests that speech praxis depends on *sensorimotor networks* underlying speech motor control, which are at least partially shared by vSTM tasks like digit span. That is, acute disruption to shared parts of these neural networks is reflected in both AOS and impaired vSTM. Because these networks involve a constellation of motor- and sensory-related regions, and because these deficits can, presumably, be caused by damage to different components of these networks, the brain region(s) implicated in any one study of AOS or vSTM will be dependent on the peculiarities of sample distribution. Furthermore, in the case of chronic stroke studies, the brain regions found to be associated with the deficits will depend on the degree of recovery of the participants studied—which may depend on lesion size, time post-onset, or even individual variability in behavioral and neuronal compensatory processes (Jarso et al., 2013). In fact, AOS or impaired vSTM might resolve more quickly than the other, even if they rely on overlapping networks of neural regions. For example, tasks of vSTM might be more “difficult”—might tax the shared sensorimotor network we have identified more than speech praxis—such that speech articulation might recover despite persistent impairment in vSTM. These complexities have resulted, we suggest, in a murky picture of the neural basis of speech praxis and vSTM. The present study avoided some of these complications by: (1) employing a fairly large sample size; (2) in an acute stage of disruption, thus precluding recovery and compensatory processes; and (3) using a sensitive measure of tissue dysfunction (infarct plus hypoperfusion). Using this approach, we conclude that AOS reflects a disruption of a relatively lower-level of sensorimotor cortical control of speech involving predominantly *somatosensory*-motor circuits that are also required for vSTM tasks (such as digit span), rather than auditory-motor circuits, which are implicated in higher-level motor control. The circuits required for speech praxis overlap with those required for vSTM (perhaps the articulatory rehearsal component) in pars opercularis portion of Broca’s area, premotor cortex, and motor cortex in the precentral gyrus.

## Conflict of interest statement

The authors declare that the research was conducted in the absence of any commercial or financial relationships that could be construed as a potential conflict of interest.
